# The Value of MRI in Distinguishing Subtypes of Lipomatous Extremity Tumors Needs Reassessment in the Era of MDM2 and CDK4 Testing

**DOI:** 10.1155/2018/1901896

**Published:** 2018-03-19

**Authors:** Sean Ryan, Julia Visgauss, David Kerr, Joshua Helmkamp, Nicholas Said, Emily Vinson, Patrick O'Donnell, Xuechan Li, Sin-Ho Jung, Diana Cardona, William Eward, Brian Brigman

**Affiliations:** ^1^Duke University Hospital, 2301 Erwin Rd., Durham, NC 27710, USA; ^2^Markey Cancer Center, University of Kentucky, 800 Rose St., Lexington, KY 40508, USA

## Abstract

**Introduction:**

Extremity lipomas and well-differentiated liposarcomas (WDLs) are difficult to distinguish on MR imaging. We sought to evaluate the accuracy of MRI interpretation using MDM2 amplification, via fluorescence in-situ hybridization (FISH), as the gold standard for pathologic diagnosis. Furthermore, we aimed to investigate the utility of a diagnostic formula proposed in the literature.

**Methods:**

We retrospectively collected 49 patients with lipomas or WDLs utilizing MDM2 for pathologic diagnosis. Four expert readers interpreted each patient's MRI independently and provided a diagnosis. Additionally, a formula based on imaging characteristics (i.e. tumor depth, diameter, presence of septa, and internal cystic change) was used to predict the pathologic diagnosis. The accuracy and reliability of imaging-based diagnoses were then analyzed in comparison to the MDM2 pathologic diagnoses.

**Results:**

The accuracy of MRI readers was 73.5% (95% CI 61–86%) with substantial interobserver agreement (*κ*=0.7022). The formula had an accuracy of 71%, which was not significantly different from the readers (*p*=0.71). The formula and expert observers had similar sensitivity (83% versus 83%) and specificity (64.5% versus 67.7%; *p*=0.659) for detecting WDLs.

**Conclusion:**

The accuracy of both our readers and the formula suggests that MRI remains unreliable for distinguishing between lipoma and WDLs.

## 1. Introduction

Lipomas are the most common soft tissue tumor and, unless symptomatic, do not require surgical excision or formal surveillance when the provider is confident in the diagnosis. However, the difference between a lipoma and well-differentiated liposarcoma (WDL) is often difficult to determine based solely on imaging [1–3]. Well-differentiated liposarcomas of the extremities have low metastatic potential and are now also commonly referred to as atypical lipomatous tumor (ALT), reflecting their benign biologic behavior relative to WDLs of the mediastinum or retroperitoneum [4]. However, given the potential for de-differentiation and conversion to a higher grade liposarcoma, excision of these lesions is recommended [2, 4, 5]. Thus, the distinction between lipoma and WDL/ALT is important, as asymptomatic lipomas need no treatment or follow-up. However, WDL/ALT of the extremity is appropriately treated with surgical excision and postoperative surveillance. Nevertheless, the ability to distinguish between WDL/ALT and benign lipomas using only MRI remains a diagnostic challenge.

**Figure 1:**
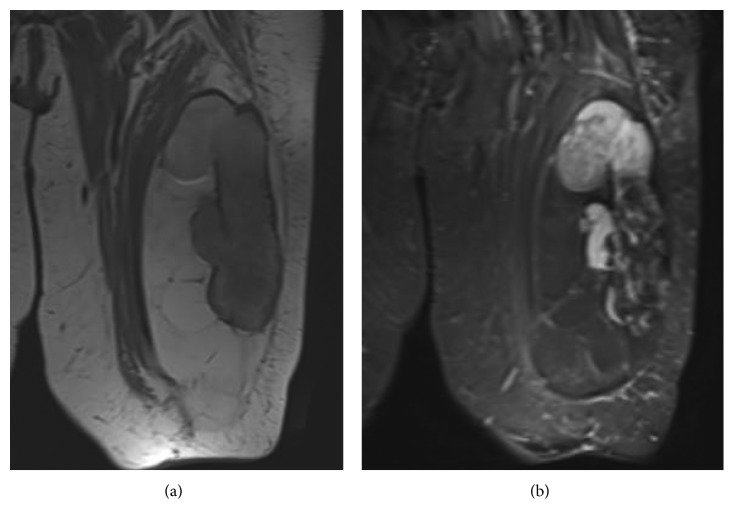
Coronal T1 (a) and STIR (b) MRI of a 30 cm diameter ALT/WDL confirmed by MDM2 FISH which is deep to fascia, contains foci of T2 enhancement, heterogeneity, nodularity, and internal cystic changes.

Numerous imaging features on MRI have been reported to facilitate differentiation between these two entities. Location deep to fascia, septations >2 mm thick, heterogeneity, foci of high T2 signal, diameter >5 cm, stranding, nodularity, and cystic changes within the tumor have been reported as being more common in ALT/WDLs than in lipomas [1, 2, 6, 7]. A representative MR image of an ALT/WDL demonstrating common concerning features is shown in Figure 1. However, these features have not allowed experts to reliably identify ALT/WDLs, and this uncertainty may lead to unnecessary patient concern and more invasive management.

Gaskin and Helms previously reported an accuracy of 83% in predicting the pathologic diagnosis based on MRI, and noted that when a lesion was suspicious for ALT/WDLs, it was more likely (64%) to represent a benign lipoma [2] after final pathology. O'Donnell and colleagues similarly compared MRI evaluation between radiologists and orthopaedic oncologists, and found an accuracy of 69% in distinguishing lipoma versus ALT/WDLs, with no difference across specialty [1]. These studies were performed using the World Health Organization (WHO) pathologic criteria for diagnosis, and both recognized the need for a reproducible method of determining the diagnosis without an invasive surgical intervention.

The difficulty identifying these tumors accurately with imaging was rendered even more complex by the fact that the pathologic criteria for diagnosis were relatively subjective. In recent years, the gold standard for accurate pathologic diagnosis has evolved with the discovery of murine double minute 2 (MDM2) gene amplification present in all ALT/WDLs. While most ALT/WDLs may be correctly diagnosed histologically, the atypia required may be focal and missed on biopsy, or under/overinterpreted. MDM2, however, is consistently amplified and its detection using fluorescence in situ hybridization (FISH) has become the new gold standard for diagnosis [3, 8]. Using this new diagnostic criterion, some tumors previously regarded as lipoma are now known to be ALT/WDLs and vice versa. This calls into question prior studies on the accuracy of MRI, as the diagnosis based on the WHO histologic criteria may have been incorrect. A recent study by Wang and colleagues constructed a scoring system for differentiating lipomas from liposarcomas utilizing MRI findings. However, this study also utilized the WHO histologic criteria without assessment of MDM2 amplification, and thus the resultant scoring system also requires further review [7].

To our knowledge, there is no study in the literature comparing the accuracy of MRI diagnosis to the new gold standard for pathologic diagnosis, MDM2. Therefore, the purpose of this study is (1) to evaluate the ability of experienced readers of MR imaging to distinguish between lipoma and ALT/WDLs in the era of MDM2 FISH, (2) to evaluate the agreement of MRI interpretations amongst experienced readers, (3) to evaluate the utility of the diagnostic formula proposed by Wang et al., and to determine whether or not it is able to outperform the interpretation of fellowship-trained readers, and (4) to determine which MRI characteristics, if any, are most predictive of the diagnosis of ALT/WDLs. Our hypothesis was that there would be an increase in the accuracy of MR imaging diagnosis given the new pathologic criteria. We further hypothesized that the weighted scoring system would provide the most accurate and reproducible diagnosis compared to expert readers by eliminating the inherent bias of readers to “overdiagnose” ALT/WDLs as shown in prior studies [1, 2].

## 2. Materials and Methods

This study was performed in collaboration with radiology, orthopaedic oncology, and surgical pathology. The cohort was retrospectively collected from the institutional database and electronic medical records were reviewed for patient demographics, available MRI, and MDM2 pathologic diagnosis. Patients with extremity lesions superficial to fascia and pathologic diagnosis based on WHO criteria were excluded from the study. Lesions outside of the extremity were excluded in part due to the subspecialties of the readers (orthopaedic oncology and musculoskeletal radiology). However, they were also excluded because WDL located in the mediastinum and retroperitoneum behave differently than those in the extremities. Following exclusion, 49 patients with deep extremity lipomas or ALT/WDLs remained.

Each patient's MRI was interpreted by two fellowship-trained orthopaedic oncologists and two fellowship-trained musculoskeletal radiologists who were blinded to the final diagnosis. All MR images reviewed contained T1-weighted and T2-weighted or STIR sequences for evaluation, and all MRI sequences were made available for reviewers at the time of the study. All four reviewers independently interpreted the images simultaneously, without time limitations, and were permitted to make measurements and analyze any desired sequence.

Readers were surveyed for each case on whether margins were well or poorly defined, tumors were homogeneous or heterogeneous, if there was stranding or nodularity, if thick septa >2 mm were present, or if there were cystic changes or foci of high T2 signal. They were then asked to make a final imaging diagnosis of lipoma or ALT/WDL. Each categorical variable, including the final diagnosis, was recorded prospectively while interpreting the MR imaging.

Cohen's Kappa coefficient were used to determine the interobserver agreement/reliability for the diagnosis and each categorical variable (margins, homogeneity, stranding, nodularity, thickened septa, internal cystic change, foci of high T2 signal, and final imaging diagnosis) for all four expert observers. Additionally, the sensitivity, specificity, accuracy, positive predictive value (PPV), and negative predictive value (NPV) of the imaging diagnosis by the expert observers were calculated based on the gold standard MDM2 diagnosis.

Next, we used the reviewer's responses to predict the diagnosis using the formula published by Wang et al. *Z* = 10*X*_1_ + *X*_2_ + 12*X*_3_ + 15*X*_4_ + 10*X*_5_, where *X*_1_ is gender (0 = female; 1 = male), *X*_2_ is tumor diameter (in cm), *X*_3_ is tumor depth (0 = superficial to fascia; 1 = deep to fascia), *X*_4_ is the presence of a septum or nodule (0 = absent septum or nodule; 1 = septum >2 mm or nodule >1 cm), and *X*_5_ is internal cystic change (0 = no, 1 = yes). All tumors were deep to fascia, meaning *X*_3_ was 1 for all patients included. *X*_4_ and *X*_5_ were determined after all lesions had been reviewed and were based on the majority opinion of the reviewers (i.e., if 2 or more expert observers felt there was a septum, nodule, or internal cystic change, then it was considered present for the formula). Of note, no reviewers had the formula available at the time of their MRI interpretation. A Z score of >35 was considered consistent with ALT/WDL as described by Wang et al., who reported a 100% negative predictive value in their study. Therefore, 35 was used as the cutoff for testing their formula in this study [7]. All diagnoses by the expert observers were subsequently grouped, and compared against the diagnoses provided by the formula, using a 2-sample paired binomial test. A *p* value < 0.05 was considered statistically significant.

Lastly, we used a stepwise regression model to select significant imaging predictors associated with a diagnosis of ALT/WDL. A significance level of 0.1 was required to allow a proposed imaging characteristic into the model.

## 3. Results

Of the 49 patients included, final pathologic diagnosis was ALT/WDLs for 18 patients and lipoma for 31 patients. There were six spindle cell lipomas and one lipoma with osseous metaplasia included in the lipoma group. Pathologic diagnosis was determined by MDM2 FISH for 44 patients and by immunohistochemistry for MDM2 and CDK4 for five patients.

Experienced readers of MR images were unable to accurately and reliably distinguish between lipoma and ATL/WDL on MR imaging. Collectively, the readers had an accuracy of 73.5% based on 2 or more readers predicting ALT/WDL. Accuracy ranged from 73.5 to 79.6% for individual observers. Expert readers showed an 83% sensitivity, 67.7% specificity, 73.5% accuracy, 60% PPV, and 87.5% NPV for interpreting the MRI for ALT/WDL when compared to the final pathologic diagnosis (Table 1).

The agreement of MR interpretation between readers was variable for each imaging characteristic; however, agreement was substantial when choosing a final diagnosis. Interobserver reliability for each imaging characteristic is shown through use of Cohen's kappa coefficient (Table 2) with foci of high T2 signal, nodularity, and final diagnosis showing the most interobserver agreement. If the final diagnosis was lipoma, MR interpreters collectively chose the correct diagnosis 68% (21/31) of the time, whereas if the final diagnosis was ALT/WDL, the correct diagnosis was interpreted for 83% (15/18). There was no significant difference in accuracy comparing expert observers against each other or across subspecialty (orthopaedic oncology versus musculoskeletal radiology). For the final diagnosis, there was 100% concordance across the four interpreters in 37/49 cases, 75% concordance in 5/49 cases, and 50% concordance in 7/49 cases.

During investigation of the formula proposed by Wang et al. using their advocated cutoff score of 35 (>35 being considered a ALT/WDLs), we found the formula to be less accurate than previously described. Using this threshold, the formula had an accuracy of 71% with sensitivity 83%, specificity 64.5%, PPV 58%, and NPV 87%. There was no significant difference in sensitivity (*p*=1.000), specificity (*p*=0.659), accuracy (*p*=0.708), PPV (*p*=0.683), or NPV (*p*=0.920) between the formula and the expert interpreters. If the pathologic diagnosis was lipoma, the formula correctly predicted the diagnosis (score ≤ 35) only 58% (18/31) of the time, whereas if the pathologic diagnosis was ALT/WDL, the formula correctly predicted the diagnosis (score > 35) for 83% (15/18) of cases.

Lastly, we employed a stepwise variable selection procedure to determine which MRI features were most associated with the diagnosis of ALT/WDL. A 10% significance level for both selection and deletion was used to identify imaging characteristics that were significantly correlated with the final pathologic diagnosis. This analysis is shown in Table 3, and only foci of high T2 signal remains in the model following selection, indicating that it is most strongly associated with the final diagnosis. Foci of high T2 signal was present in 16/18 (88.9%) ALT/WDLs and in 20/31 (64.5%) lipomas.

## 4. Case Illustrations

**Figure 2:**
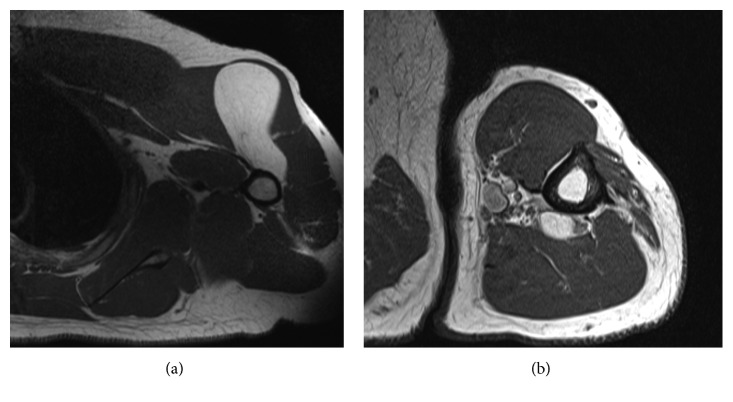
(a) Axial T1 MR of left shoulder with 12 cm lipomatous tumor. Formula and expert observers predicted benign lipoma, while initial pathology was concerning ALT/WDL. Lack of MDM2 amplification with FISH confirmed a diagnosis of benign lipoma. (b) Axial T1 MR of the left arm with 2 cm lipomatous tumor. Formula and expert observers predicted benign lipoma, which was later confirmed by lack of MDM2 amplification through FISH after initial pathology was concerning ALT/WDL.

Three cases from this cohort were selected in order to highlight the significance of MDM2 FISH and the difficult agreement between MRI interpretation and pathology. The first is the case of a 42-year-old male with a 12 cm lipomatous tumor in the left shoulder. Based on MR imaging, 4/4 expert observers, in addition to the formula (score of 34), predicted the lesion to be a benign lipoma. Initial pathology was concerning ALT/WDL, which was subsequently overturned following MDM2 FISH because there was no amplification of the MDM2 gene. The second is the case of a 69-year-old male with a 2 cm lesion in the left arm. Again, all four expert observers and the formula (score of 24) predicted the lesion to be a benign lipoma, but pathology was concerning ALT/WDL. Following MDM2 FISH, the pathologic diagnosis was subsequently confirmed to be a benign lipoma, increasing the accuracy of our readers and the formula (Figure 2).

**Figure 3:**
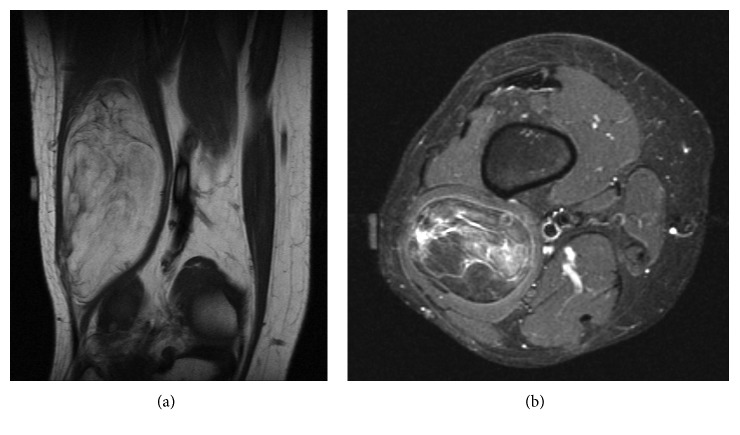
(a) Coronal T1 and (b) axial STIR MR images of the right thigh lipomatous tumor. Expert observers interpreted the lesion as heterogeneous, containing stranding, and having foci of high T2 signal. All four readers interpreted the tumor as ALT/WDL in addition to initial pathology interpreted as ALT/WDL. MDM2 amplification during FISH analysis confirmed benign lipoma on two separate occasions, illustrating the variability that can be seen within simple lipomas.

The final case is a 51-year-old female with a 10 cm lipomatous lesion of the right thigh. All four expert observers predicted the lesion to be an ALT/WDL, while the formula (with score of 22) predicted the lesion to be a benign lipoma. Initial pathology report was read as ALT/WDL. Subsequent MDM2 FISH was negative for gene amplification, and given the histologic concern for atypia, the FISH was again repeated and found to again be negative, confirming the diagnosis of lipoma and changing the pathologic diagnosis (Figure 3).

## 5. Discussion

Liposarcomas represent approximately 20% of all soft tissue sarcomas and are divided into various subtypes including atypical lipomatous tumors/well-differentiated liposarcomas (ALT/WDLs), de-differentiated liposarcoma, myxoid liposarcoma, round cell liposarcomas, and pleomorphic liposarcomas. ALT/WDLs are often difficult to distinguish from benign lipomas on MRI [1, 2, 6, 9, 10] and have different treatments as well as prognosis. On MRI, ALT/WDLs are known for having thick septa, lack of capsule with less well-defined margins, nodularity, internal cystic changes, heterogeneity, and enhancement on T2-weighted imaging. Meanwhile, lipomas are characteristically homogeneous and encapsulated lesions composed of predominantly mature adipose tissue. However, they may contain thin enhancing septa, be unencapsulated, or have heterogeneity due to regions of fat necrosis, infarction or nonfatty tissue, and creating concern for ALT/WDLs on MRI interpretation [1, 2, 6, 9].

The importance of distinguishing between lipoma and ALT/WDLs preoperatively is well recognized. For many providers, ALT/WDLs receive wide local excision rather than marginal excision to decrease the risk of local recurrence. Following surgery for ALT/WDLs, recurrence rates vary from 13.9 to 69% [2, 11]. Lucas and colleagues, however, found that there was a 60% local recurrence rate if extremity tumors were treated with marginal excision, which decreased to 11% when treated with wide local excision [5]. The appropriate treatment remains debated, however, as some advocate for marginal excision with or without radiation therapy given low rates of local recurrence and the morbidity associated with wide resection [4, 11, 12]. Importantly, many of these studies on local recurrence rates were not based on molecular analysis for diagnosis, and therefore, may have had inaccurate categorization of lipomatous tumors, leading to falsely low recurrence rates if lipomas were regarded as ALT/WDLs. Furthermore, ALT/WDLs have the potential for delayed malignant de-differentiation and subsequent metastasis. Due to this potential for delayed de-differentiation as well as the higher local recurrence rates compared to lipomas, the importance of continued surveillance postoperatively regardless of surgical margin is well established [1, 2, 6, 11–14].

Previous publications have shown relatively poor accuracy for MRI predicting the pathologic diagnosis. O'Donnell and colleagues found a 69% overall accuracy for expert observers predicting the pathologic diagnosis based on the WHO criteria, while Gaskin and Helms found an 83% specificity and a 38% PPV [1, 2]. These studies noted a propensity for MRI interpreters to overdiagnose ALT/WDLs, leading to unnecessary patient worry and more invasive surgical intervention. In an effort to create a more objective interpretation of imaging findings, Wang and colleagues devised a scoring system based on MR imaging features which were found to be correlated to the final pathologic diagnosis in their series [7]. In their study, all ALT/WDLs had a *Z* score of >35 and 30/34 benign lipomas had a *Z* score of ≤35. Therefore, they proposed this scoring system as a potential alternative to invasive biopsies for preoperative decision-making. These authors, similar to others looking at the accuracy of MRI, utilized the WHO criteria for final pathologic diagnosis rather than MDM2 amplification. Therefore, some tumors may have been misclassified, requiring revalidation of the scoring system.

Not only can it be difficult to determine lipoma from ALT/WDLs for the radiologist, it can also pose a challenge to the pathologist. Histologically, the degree of atypia may be overestimated or confounded by fat necrosis, especially in borderline cases [3, 8]. Furthermore, tumor heterogeneity can lead to sampling error and inaccurate histologic diagnosis. This difficulty can be seen in our three case examples above, which all had concerning features on initial pathology. With advances in modern molecular analysis, however, the gold standard for pathologic diagnosis of ALT/WDLs has changed. Specifically, murine double minute 2 (MDM2) has been found to be amplified in all ALT/WDLs [10]. The use of immunohistochemistry (IHC) for MDM2 and CDK4 has been proposed as a more cost-effective solution for determining the diagnosis and is widely available, while FISH is typically only used at tertiary referral centers. However, for borderline cases, MDM2 FISH has been shown to be required for accurate pathologic diagnosis given the potential for sampling error on biopsy and subjective interpretation of MDM2 immunohistochemistry [8, 15]. Meanwhile, MDM2 FISH has been shown to have 100% sensitivity and specificity even on core needle biopsy [16]. This new criterion was the focus of the current study. The goal was to reassess the ability of expert observers to distinguish lipoma from ALT/WDL on MRI, given the increased accuracy of pathologic diagnosis of these tumors using MDM2 as the gold standard. As stated previously, 44 patients had final pathology determined by MDM2 FISH, and 5 patients had IHC for final diagnosis.

Our series showed agreement amongst expert interpretation of MRI for both orthopaedic oncologists and musculoskeletal radiologists. Collectively, our expert observers had an accuracy of 73% for predicting the final pathologic diagnosis, which is consistent with prior reports. A PPV of 60% and NPV of 87.5% indicated a tendency to overdiagnose ALT/WDL, as reported by prior authors [1, 2]. The formula proposed by Wang et al. showed an accuracy of 71.4% with a 57% PPV and 87% NPV, slightly underperforming our expert observer”s interpretation. This difference, however, was not statistically significant. Unlike the study by Wang et al., all of the tumors included in our study were deep to fascia, making it more difficult to discern lipoma from ALT/WDLs. This may account for the reduced accuracy of the formula seen in our cohort.

Of the various MRI features tested, foci of high T2 signal intensity had the highest correlation with a diagnosis of ALT/WDL, unlike the study by Wang et al., which found internal cystic change, nodules, and thick septum to be more predictable factors [7]. However, it is important to note that foci of high T2 signal were also present in 64.5% of benign lipomas. This highlights the variability of these tumors and reveals a multitude of MRI findings based on a representative cohort. This can be seen in other areas throughout the current literature; for example, O'Donnell and colleagues focused on stranding, nodularity, and size as the determinant factors for diagnosis [1].

We continue to understand the utility of MR imaging for the generation of a differential diagnosis and preoperative planning. However, based on our results, we do caution readers that MRI should not be used in isolation for diagnosis. At this stage, despite numerous advances, we feel that further study is required for alternative and less invasive means of diagnosis to guide appropriate management of these lesions preoperatively. Furthermore, we recognize that MDM2 FISH (or MDM2/CDK4 IHC) may not be available at all institutions, especially given its high cost. While all lesions do not necessarily require MDM2 FISH, we agree with Clay and colleagues that when lesions are recurrent, deep and >10.0 cm, have equivocal atypia, and are concerning on MR imaging, they warrant investigation with MDM2 for definitive diagnosis [3].

## 6. Conclusion

While MDM2 FISH has affected the confidence with which pathologists can diagnose ALT/WDLs, it remains difficult for expert observers to distinguish them from benign lipomas on imaging. Based on the data presented here, expert readers of MRI have an accuracy of 73% in distinguishing lipoma from ALT/WDL, which is consistent with prior reports. The use of MDM2 FISH for pathologic diagnosis, while more reliable than the WHO criteria, has not changed the accuracy of MRI interpretation. Furthermore, the proposed scoring system by Wang et al. was found to have less utility than previously reported, with an accuracy of 71%. Therefore, while MRI is an important screening tool for differentiating these lesions, pathologic confirmation with MDM2 FISH is still required for diagnostic certainty.

## Figures and Tables

**Table 1:** Expert observer grouped interpretation of MRI compared to final pathologic diagnosis.

	Value	95% CI lower bound	95% CI upper bound
Sensitivity	0.8333	0.6612	1
Specificity	0.6774	0.5129	0.8420
Accuracy	0.7347	0.6111	0.8583
Positive predictive value (PPV)	0.6000	0.4080	0.7920
Negative predictive value (NPV)	0.8750	0.7427	1

**Table 2:** Interobserver reliability amongst four expert readers of MRI for commonly reported imaging characteristics.

	Margins WD versus PD	Homogeneous	Stranding	Nodularity	Thickened septa	Internal cystic change	Foci of high T2 signal	Imaging diagnosis
Kappa	0.2331	0.6122	0.4552	0.7673	0.5890	0.3333	0.7959	0.7022
*p* value	0.0001	<0.0001	<0.0001	<0.0001	<0.0001	<0.0001	<0.0001	<0.0001

**Table 3:** Imaging parameter in stepwise model selection. Significance coefficient of 0.1 was required to remain in the model.

Parameter	Coefficient	OR	95% CI for OR		*p* value
			Lower	Upper	
*First selection*					
Intercept	−1.091				0.154
Margins (WD versus PD)	−0.375	0.472	0.097	2.290	0.352
Homogeneous (yes versus no)	−0.896	0.167	0.010	2.720	0.209
Stranding (yes versus no)	0.056	1.118	0.079	15.732	0.934
Nodularity > 1 cm (yes versus no)	0.001	1.002	0.155	6.486	0.998
Thickened septa > 2 mm (yes versus no)	−0.620	0.289	0.019	4.290	0.367
Internal cystic change (yes versus no)	−0.186	0.689	0.036	13.098	0.805
Foci of high T2 signal (yes versus no)	1.142	9.822	0.906	106.436	0.060
*Second selection*					
Intercept	−0.9639				0.0215
Foci of high T2 signal (yes versus no)	1.3386	14.545	2.811	75.270	0.0014
